# Upper Respiratory Tract Microbiome and Otitis Media Intertalk: Lessons from the Literature

**DOI:** 10.3390/jcm9092845

**Published:** 2020-09-02

**Authors:** Francesco Folino, Luca Ruggiero, Pasquale Capaccio, Ilaria Coro, Stefano Aliberti, Lorenzo Drago, Paola Marchisio, Sara Torretta

**Affiliations:** 1 Department of Pathophysiology and Transplantation, University of Milan, 20122 Milan, Italy; ilaria.coro@policlinico.mi.it (I.C.); stefano.aliberti@unimi.it (S.A.); paola.marchisio@unimi.it (P.M.); 2 Pediatric Highly Intensive Care Unit, Fondazione IRCCS Ca’ Granda Ospedale Maggiore Policlinico, 20122 Milan, Italy; luca.ruggiero@policlinico.mi.it; 3 Department of Otolaryngology and Head and Neck Surgery, Fondazione IRCCS Ca’ Granda Ospedale Maggiore Policlinico, 20122 Milan, Italy; pasquale.capaccio@unimi.it (P.C.); sara.torretta@unimi.it (S.T.); 4 Department of Biomedical Surgical Dental Science, University of Milan, 20122 Milan, Italy; 5 Internal Medicine Department, Respiratory Unit and Adult Cystic Fibrosis Center, Fondazione IRCCS Ca’ Granda Ospedale Maggiore Policlinico, 20122 Milan, Italy; 6 Laboratory of Clinical Microbiology, Department of Biomedical Science for Health, University of Milan, 20122 Milan, Italy; lorenzo.drago@unimi.it; 7 Department of Clinical Sciences and Community Health, University of Milan, 20122 Milan, Italy

**Keywords:** otitis media, microbiota, upper respiratory tract, adenoid, middle ear, microbiota axes

## Abstract

Otitis media (OM) is one of the most common diseases occurring during childhood. Microbiological investigations concerning this topic have been primarily focused on the four classical otopathogens (*Streptococcus pneumoniae*, *Haemophilus influenzae*, *Moraxella catarrhalis* and *Streptococcus pyogenes*) mainly because most of the studies have been conducted with culture-dependent methods. In recent years, the introduction of culture-independent techniques has allowed high-throughput investigation of entire bacterial communities, leading to a better comprehension of the role of resident flora in health and disease. The upper respiratory tract (URT) is a region of major interest in otitis media pathogenesis, as it could serve as a source of pathogens for the middle ear (ME). Studies conducted with culture-independent methods in the URT and ME have provided novel insights on the pathogenesis of middle ear diseases through the identification of both possible new causative agents and of potential protective bacteria, showing that imbalances in bacterial communities could influence the natural history of otitis media in children. The aim of this review is to examine available evidence in microbiome research and otitis media in the pediatric age, with a focus on its different phenotypes: acute otitis media, otitis media with effusion and chronic suppurative otitis media.

## 1. Introduction

The human microbiota consists of ecological communities of commensal, symbiotic and pathogenic microorganisms that colonize several body sites, as the gastrointestinal tract, respiratory system, oral cavity, skin and female reproductive system [1]. In past years, microbiological investigations have been predominantly conducted with culture-dependent methods, therefore many sites in the human body have been considered sterile until recently. However, the introduction of culture-independent techniques has allowed investigation of entire bacterial communities [2], leading to a better comprehension of the role of resident flora in health and disease. These microorganisms and their products play indeed a critical role in the regulation of many homeostatic processes, including immune response and inflammation [3] and defense against pathogens [4]. A diseased alteration in the composition of these bacterial communities, defined dysbiosis, can therefore lead to many pathological conditions, including infections [5].

Most of these studies have been conducted with a marker gene analysis based on a broad-range PCR, using primers that target a segment of the 16SrRNA gene, a highly conserved region contained in bacterial genomes. This method, combined with next-generation sequencing technologies, permits the simultaneous characterization of an entire community [6]. This approach allows a fast and cost-effective analysis that provides a low-resolution view of a microbial community. However, there are also some limitations that should be taken into account when interpreting data derived from these studies: it is not possible to determine whether taxa detected are alive or dead, active or inactive, thus there is limited functional information; it is susceptible to over amplification bias, especially with low biomass samples such as middle ear fluid; as a short segment of 16SrRNA gene is amplified and sequenced, taxonomic resolution is usually limited to family or genus level; there is great variability depending on technical aspects as region selection, amplicon size, sampling, storage, sequencing approach, and bioinformatic analysis. Full-gene 16S rRNA gene sequencing and metagenome and metatrascriptome analyses may overcome some of these limitations but are less adopted as they are relative expensive and complex to perform [7]. Middle ear infections and diseases are widespread in pediatric age. Acute otitis media (AOM) is the most common bacterial infection in childhood [8] and the leading cause of antibiotic prescription in pediatric patients [9]; similarly, otitis media with effusion (OME) is prevalent in the first years of life, as up to 80% of children suffer from one or more episodes by 10 years of age; however, it should be considered that the prevalence of OME varies across population and could be difficult to define accurately, as this condition is often asymptomatic [10].

The upper respiratory tract (URT) is a region of major interest in otitis media pathogenesis: According to the Pathogen Reservoir Hypothesis (PRH), the adenoid pad serves as a source of pathogens that can grow in this region and further spread to the respiratory system and middle ear, leading to infections and diseases [11,12,13,14].

The URT extends from the nostrils to the portion of the larynx above the vocal cords and harbors the highest bacterial density in the whole respiratory system [15]; however, these bacterial communities have been studied with more effort and from an ecological perspective only in recent years, after the introduction of culture-independent techniques [16].

Scientific interest has been focused on the comprehension of the characteristics of a healthy URT microbiota and the mechanism that guarantees its balance, as mounting evidence shows that resident bacteria are able to inhibit colonization and growth of otopathogens [14,17,18]. Those microorganisms that are essential in maintaining balance and function of a bacterial community are defined keystone species (see Table 1 for definitions of common terms used in microbiota analysis). In the URT, *Dolosigranulum* spp. and *Corynebacterium* spp. have been identified as potential keystone species, as they have been associated with respiratory health and exclusion of otopathogens in several studies [19,20,21,22].

Reconstitution of healthy microbial communities through administration of probiotics for the prevention of middle ear diseases in children is a topic of major clinical and scientific interest. Several trials have been conducted, but results lack consistency [23,24]. Deepening our knowledge on the physiological features of the URT microbiota and understanding how modifications in its balance relate to the pathogenesis of otitis media could be of remarkable importance in developing probiotic therapies. Furthermore, middle ear microbiota involvement in this field has been gaining interest in recent years, although less studies are available in comparison with URT microbiota, due to the different feasibility in collecting samples.

The aim of this review is to examine evidence available in microbiome research on otitis media in children. We will describe the most important factors that impact on microbiota development in the first years of life and that could influence the natural history of otitis media; then, we will focus on otitis media phenotypes and discuss evidence available on URT and middle ear microbiome in different diseases.

## 2. Methods

The research was conducted on the PubMed database, including all evidences available until April 2020. MeSH terms as “otitis media”, “microbiota”, “child”, “child, preschool” and “infant” were used. More articles were included combining the keywords “microbiota” and “microbiome” with terms as “acute otitis media”, “otitis media with effusion”, “chronic otitis media”, “adenoid”, “adenotonsillar”, “nasopharyngeal”, “middle ear”.

**Figure 1:**
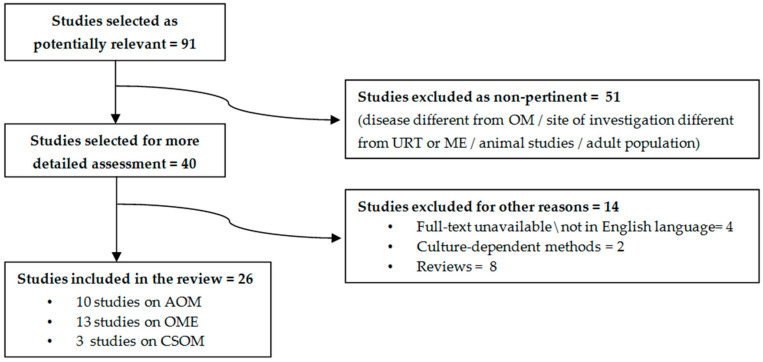
Search strategy conducted for this review. Legend: OM: Otitis media. AOM: Acute otitis media. OME: Otitis media with effusion. CSOM: Chronic suppurative otitis media. ME: Middle Ear. URT: Upper respiratory tract.

A total of 91 potentially relevant studies were identified through this search strategy. After title and abstract analysis, 51 studies were excluded as non-pertinent, according to the following criteria: disease different from OM; site of investigation different from URT or ME; adult population; studies conducted on animals were also excluded, as the main focus of this review was to discuss evidence available in children. A total of 40 remaining articles were then selected for more detailed assessment, and 14 investigations were further excluded in this phase (see Figure 1 for more details on methods).

After this process, 26 studies were included in this review: 10 studies on acute otitis media (AOM, 1321 subject enrolled in all studies), 13 studies on otitis media with effusion (OME, 501 subjects enrolled in all studies), 3 studies on chronic suppurative otitis media (CSOM, 217 subjects enrolled in all studies).

## 3. Environmental Factors and Microbiota Development in the First Years of Life

The microbial communities that colonize the human organism are dynamic and change throughout life under the effect of several environmental factors, but infancy and early childhood represent the critical period in shaping their composition [25,26]. These external factors can impair the homeostatic functions mediated by the microbiota, leading to immediate consequences or impacting the health status in the later stages of life [27]. This is particularly evident for the URT microbiota, as this region is interconnected with middle ear, lower respiratory tract, and gastrointestinal tract, and represents the interface between these systems and the external environment.

Immediately after birth, in the first hours of life, the URT in healthy neonates becomes colonized by microorganisms of maternal origin [28]. Niche differentiation starts in the first week of life, with a predominance of *Staphylococcus* spp., followed by an enrichment of *Corynebacterium*, *Dolosigranulum*, and *Moraxella* [29].

The first months of life are of remarkable importance in the development of URT microbial communities and their composition: Biesbroek et al. described eight distinct microbiota profiles in the URT of healthy infants, showing that a distinct bacterial profile could be identified by the sixth week of life; moreover, this early bacterial colonization plays a pivotal role in the stability of microbial communities: profiles dominated by *Moraxella* and *Dolosigranulum/Corynebacterium* are associated with a stable microbiota and with lower rates of respiratory infections in later stages of life, while less stable profiles are associated with high abundance of *Haemophilus* and *Streptococcus* [30].

Theo et al. confirmed the role of *Corynebacterium* and found a positive role of *Alloiococcus* in the first year of life in the development of URT microbial communities; moreover, authors reported data on nasopharyngeal (NP) microbiota in children with respiratory diseases, concluding that some *Moraxella* spp. were associated with an increased risk of disease rather than respiratory health [31].

Several environmental factors, discussed below, can influence the shaping of the URT microbiota composition in the first years of life.

### 3.1. Delivery Route

As it is generally known, children born by caesarian-section (C-section) suffer from a higher incidence of respiratory illness and morbidity in comparison with children born by vaginal delivery [32,33].

In one of the first reports concerning nasopharyngeal microbiota and route of delivery, swabs from different body sites were collected from healthy neonates immediately after birth: Authors found that undifferentiated microbial communities in vaginally delivered children were similar to maternal vaginal microbiota, while those who were born by C-section had microbial communities resembling maternal skin surface [28].

A subsequent longitudinal study on this theme analyzed nasopharyngeal swabs collected from 102 children in the first 6 months of life, showing a predominance of bacteria previously associated to microbiome stability and respiratory health in early stages of life (*Moraxella*, *Corynebacterium*, and *Dolosigranulum*) in children born by vaginal delivery [29]. These microorganisms are likely derived from maternal skin (*Staphylococcus* and *Corynebacterium*) [34] or from vaginal tract (*Dolosigranulum*, *Staphylococcus*, or *Streptococcus*) [35].

However, by contrast, another study evidenced that differences related to delivery route are transient and disappear by six weeks of age, suggesting that the development of the microbiota in the postnatal period is more related to the body site that harbors a community [36].

### 3.2. Breastfeeding

Breastfeeding is a significant protective factor against infections [37,38]. This effect is related not only to the presence of antibacterial substances in maternal milk [39], as it is known that breastfeeding can significantly facilitate the development of a healthy microbiota.

Biesbroek et al. showed that breastfed infants develop a bacterial profile enriched by *Dolosigranulum* and *Corynebacterium* at six weeks of age in comparison with formula fed infants; moreover, *Dolosigranulum* abundance was inversely associated with wheezing episodes and a number of parental reported respiratory tract infections, even after correction for feeding type. [40].

Similar data were provided by Bosch et al.: Children who suffered from a higher number of respiratory infections had an aberrant nasopharyngeal microbiota development in the first month of life, that coincided with a prolonged reduction of *Dolosigranulum* and *Corynebacterium*; authors found that breastfeeding was an independent driver of this aberrant development, as a prolonged dominance of these bacteria was observed in breastfed infants. However, similarly to delivery route, these dissimilarities are transient and disappear around six months of age [41].

### 3.3. Antibiotic Therapy

Antibiotic therapy can significantly impair composition and balance of the microbiome [42]. This is particularly relevant in pediatric age, in which antibiotic prescription and misuse is quite common [43].

In the URT, antibiotic administration causes a reduction of the abundance of potential beneficial bacteria, such as *Dolosigranulum* and *Corynebacterium*, and an increase in *Haemophilus*, *Streptococcus*, and *Moraxella* [31]. Moreover, in children with AOM, a recent antibiotic therapy induces a reduction of *Streptococcaceae* and *Corynebacteriaceae* and an increased abundance of *Enterobacteriaceae* and *Pasturellaceae* in the URT [44]. Subsequent longitudinal studies confirmed how antibiotic treatment can induce a reduction in the abundance of potential beneficial bacteria, as *Dolosigranulum* and *Corynebacterium* [41,45].

### 3.4. Pneumococcal Vaccination

The introduction of the pneumococcal conjugate vaccination (PCV) in the pediatric population has led to an important reduction of OM episodes caused by the serotypes included in the vaccine [46]. On the other hand, the introduction of pneumococcal vaccination programs has resulted in important modifications in OM microbiology: *H. influenzae* has become the most common otopathogen and OM episodes caused by *M. catarrhalis* have become more frequent; moreover, serotypes not included in PCVs have been more frequently identified as causative agents of diseases [47,48].

These findings suggest that the introduction of PCVs might have induced modifications in the composition of the microbial communities in the respiratory system. However, evidence supporting these findings has been focused prevalently on otopathogens, while studies conducted with high-throughput methods and looking at whole bacterial communities in the URT are lacking and show conflicting results.

In one of the first investigations on the whole NP microbiota and AOM, Hilty et al. reported that a previous exposure to PCV-7 in children with AOM was associated with reduced abundance of commensal families (*Streptococcaceae* and *Corynebacteriaceae*) [44].

A possible influence of this vaccination on NP microbiota was later reported by Biesbroek et al. In this investigation, NP swabs were collected from healthy children who received PCV-7 and from unvaccinated children: vaccination affected the URT microbiota causing a shift in composition and structure of the bacterial community, with an increase of *Veillonella, Prevotella, Fusobacterium, Leptotrichia, Actinomyces*, *Rothia*, and non pneumococcal streptococci, in addition to an increased bacterial diversity and inter-individual variability [49].

Longitudinal data on this theme were further provided in another study conducted in Switzerland by Mika et al., who compared NP microbiota in healthy children who were vaccinated with PCV-7 or PCV-13, showing that those who received PCV-13 had a more diverse and stable URT microbiota and a lower pneumococcal carriage rate compared to those who received PCV-7 [50].

However, in contrast to these findings, other available studies suggest that PCV might not have such a relevant impact on the URT microbiota. Faezel et al. performed a randomized controlled trial in Kenya comparing NP microbiota of children who received a 10-valent pneumococcal vaccine vs. children who received Hepatitis A vaccine. In this longitudinal study, NP swabs were collected before the administration of the vaccine and after 6 months. The authors found that PCV did not cause any significant alteration in the abundance or prevalence of otopathogens [51].

Moreover, a more detailed longitudinal study conducted in Gambia analyzed NP swabs collected periodically from birth to the first year of life. Children were divided in three groups according to vaccination schedule: Two groups received two different types of PCV-7, while the third group was composed by unvaccinated children. Again, bacterial communities were comparable across groups, as there were no significant differences in richness, diversity, and composition. Interestingly, PCV-7 vaccination reduced the nasopharyngeal carriage of vaccine serotypes, but pneumococcal carriage remained high among vaccinated infants, probably because of an immediate expansion of non-vaccine serotypes [52].

Interesting data were provided by Andrade et al. in a complex investigation that compared 53 children vaccinated with PCV-10 vs. 27 unvaccinated children. The strength of this study is the integrated metagenomic and transcriptomic analysis: no difference were found in nasopharyngeal carriage rates of *S. pneumoniae*, *S. aureus*, *H. influenzae*, or *M. catarrhalis* by either transcriptomic ormetagenomics analysis, but unvaccinated children had higher metabolic rates for *S. pneumoniae*, compared to PCV-10 vaccinated children [53].

Available evidence thus suggest that PCV has a direct impact on pneumococcal carriage, which in turn might indirectly affect the whole bacterial community in the URT. However, results are conflicting: a possible explanation could be found in the variation of pneumococcal carriage rates in relation to the geographic region and socio-economic status: the effects of PCV might indeed be different while considering developed or developing countries [54].

This particular theme was investigated in a study conducted in Fiji, in which NP microbiota from two ethnic groups (iTaukei and Fijians of Indian descent) was analyzed. These groups are known to have a different carriage prevalence of *S. pneumoniae* and a different burden of pneumococcal disease, which is higher in the iTaukei population. NP swabs were collected from 132 total children belonging to the two ethnic groups that were further divided in two subgroups based on whether children had been previously vaccinated or not with PCV-7. The vaccination had no overall impact on microbial diversity or composition, but significant modifications were evident when stratifying by ethnicity: vaccinated iTaukei children had a lower relative abundance of *Streptococcus* and *Haemophilus* compared with unvaccinated ones, while vaccinated Indian descent children had a higher relative abundance of *Dolosigranulum* compared with those unvaccinated [55].

### 3.5. Smoking

Studies conducted in adult subjects suggest that active smoking impairs URT microbiota composition [56]. It is likely that similar effects involve the pediatric population; however, studies concerning active and passive smoking effects on URT microbiome in children are lacking.

## 4. Acute Otitis Media

Acute otitis media (AOM) is defined by the presence of fluid in the middle ear associated to signs and symptoms of acute infection. It affects the majority of children in the first 3 years of life and becomes recurrent in almost 50% of cases [10]. Recurrent acute otitis media (RAOM) is defined as four or more AOM episodes in one year or three or more episodes in 6 months [57].

Laufer et al. performed one of the first studies comparing NP microbiome in children with AOM to healthy children. The authors evidenced that a higher relative abundance of *Corynebacterium* and *Dolosigranulum*, in addition to *Propionibacterium*, *Lactococcus*, and *Staphylococcus*, was associated with a lower incidence of pneumococcal colonization and AOM. The same study showed that a less diverse and a less even microbiota was associated with colonization by *S. pneumoniae*, highlighting the correlation between a higher biodiversity and better outcomes [20].

These data were confirmed in a subsequent investigation conducted by the same group on 240 children aged 6 months–3 years, that evidenced that a lower biodiversity was associated with a higher colonization rate not only from *S. pneumoniae* but also from *H. influenzae* and *M. catharralis*; moreover, authors compared diversity indices between health status and during an acute upper respiratory infection (URTI), showing that biodiversity was significantly higher in healthy children than during disease [19].

These findings on biodiversity during URTI are coherent with data provided by Hilty et al. that evidenced how NP bacterial density is lower in children during an AOM episode compared with the same in healthy status. Moreover, interesting insights were provided on how the infants’ microbiota undergoes changes during an AOM episode, as the classical otopathogens predominated over commensal families (*Staphylococcaceae*, *Flavobacteriaceae*, *Carnobacteriaceae*, and *Comamonadaceae*) [44].

In 2017 Chonmaitree et al. performed a longitudinal study on 139 healthy neonates, followed since birth for the first 12 months of life or until the occurrence of the first AOM episode, collecting 971 swabs performed monthly and during an URTI or AOM. In particular, as it is known that URTI often precedes an AOM episode, authors studied the characteristics of the NP microbiome during transitional phase from URTI to AOM. Data revealed that an unstable microbiota during an URTI episode with the predominance of otopathogens were associated with the occurrence of symptomatic viral infection and with a higher risk of transition from URTI to AOM. Interestingly, otopathogens were not predominant during otherwise asymptomatic viral infections [45].

Evidence on otitis-prone children, i.e., those already suffering for RAOM were provided by Dirain et al.: Authors compared the microbial flora on adenoid tissue in a small group of subjects undergoing adenoidectomy for RAOM (*n* = 5) or obstructive sleep apnea (OSA) (*n* = 5), finding that the relative abundance of *S. pneumoniae* and *M. catharralis* was higher in the RAOM group [58].

A complex study with a higher sample size was subsequently performed on an Australian population, comparing NP microbiome of 103 healthy children vs. 93 otitis-prone children undergoing grommet insertion for RAOM, in order to identify potential protective genera. This investigation confirmed the pivotal role of *Dolosigranulum* and *Corynebacterium* in NP microbiome, as these two genera have been found to be significantly more abundant in the NP of healthy children compared with otitis-prone children. As for biodiversity, in contrast with previous findings, this study found that otitis-prone children had a significantly more diverse microbiome than controls. In addition, authors analyzed middle ear fluid (MEF) microbiome collected from children undergoing surgery from RAOM and performed a paired comparison with the NP microbiome of the same subject. Results showed that these two niches were not highly concordant: In particular, the interesting data is that *Alloiococcus* and *Turicella* have been found to be abundant in MEF but almost absent in the NP [22].

The MEF microbiome during an AOM episode was further investigated on 79 subjects aged 5–42 months. This report confirmed that the classical otopathogens are the predominant species in MEF during AOM: *S. pneumoniae* was dominant in 16% of samples, *H. influenzae* in 17%, and *M. catarrhalis* in 5.6%; moreover, *Turicell aotitidis* was detected as a clearly dominant bacteria in two samples, suggesting that it could be a rare but true causative agent; *Alloiococcus otitidis* was detected only in 3 samples; *Staphylococcus auricolaris* was predominant in two samples, but authors speculated that this finding could be related to potential contamination from the external auditory canal (EAC); however, *A. otitidis* and *T. otitidis* could be also related to EAC contamination [59].

Xu et al. compared the MEF microbiota during AOM episode to the NP microbiota analyzed on nasal wash (NW) samples: A significantly higher abundance of *A. otitidis* was detected in MEF during AOM, compared with NW in health and disease; authors concluded that the ME could harbor a resident microbiome that becomes different from NP after the onset of an infection. Moreover, NP microbiome was analyzed prior to the onset of AOM vs. at AOM onset: In line with previous data, NP microbiome during health was significantly more diverse than during AOM [60].

Paired analysis of NP and MEF microbiome during an AOM episode was subsequently performed on a larger population, collecting 286 NP swabs in children aged 0–6 years; 42/286 episodes were characterized by spontaneous tympanic membrane perforation (STMP), and thus, MEF microbiome was analyzed in these cases. Authors found that diversity was strictly related to age: in particular, older children had a higher richness and showed more personalized bacterial profiles, that develop toward the end of the sixth year of life. The transition to an adult-like microbiome appeared in children older than 3 years and was defined by an increase in *Staphylococcaceae* and *Corynebacteriaceae*. Furthermore, authors found concordance between NP and MEF microbiome when the predominant bacteria in MEF was *S. pyogenes*, *H. influenzae*, or *S. pneumoniae*. However, even this event appeared to be age-related, as the concordance between NP and MEF microbiome became weaker as children got older. Authors thus concluded that the NP microbiota does not necessarily resembles the one in ME: The URT in children with AOM serves as a moderate proxy for MEF at a very young age but becomes more diverse at a more advanced age [61].

The most frequently observed complication of AOM in clinical practice is the spontaneous tympanic membrane perforation (STMP) [62]. However, evidence on microbiota in children with history of RAOM with STMP is lacking. We believe that this condition represents a distinct phenotype of disease in otitis-prone children [63], and more effort should be directed to this category of patients, since their clinical management is often very challenging, and the most important AOM preventive measurements are often less effective [64,65,66].

Man et al. conducted a study on 94 children with tympanostomy tubes who suffered from ear discharge. In this case, authors observed a substantial concordance between paired NP and MEF microbiota, thus supporting the pathogen reservoir hypothesis: in particular, *Pseudomonas aeruginosa*, *Staphylococcus aureus*, *Streptococcus pyogenes*, *Turicella otitidis*, *Klebsiella pneumoniae*, and *Haemophilus* spp. were correlated between these two sites. *Moraxella* spp., *Streptococcus pneumoniae*, and *Corynebacterium/Dolosigranulum* were predominant in NP rather than in MEF, confirming their role as keystone bacteria of the URT; by contrast, *Turicella*, *P. aeruginosa* and *S. aureus* were strongly associated to MEF. Of interest, abundance of *Corynebacterium* and *Dolosigranulum* in NP related to a shorter course of the disease and better clinical outcomes [67].

Evidence available on AOM display that *Dolosigranulum* and *Corynebacterium* might act as potential keystone taxa in the URT, as they have been associated to a healthy status and to a lower colonization rate by otopathogens such as *S. pneumoniae*. Moreover, studies conducted on MEF identify *A. otitidis* and *T. otitidis* as possible novel otopathogens, although the theme of sample contamination from the EAC deserves major clarification.

An overview on microbiome study in AOM previously discussed is reported in Table 2.

## 5. Otitis Media with Effusion

Otitis media with effusion (OME) is defined as the presence of middle ear fluid without signs or symptoms of acute infection. It is defined chronic otitis media with effusion (COME) whether it persists for more than 3 months [10].

The first study on this topic with a high-throughput molecular approach was conducted by Liu et al., through the investigation of the microbiota of middle ear, adenoid, and tonsils in an 8-year old child with chronic middle ear effusion undergoing adenotonsillectomy and bilateral tympanic tube insertion. Middle ear microbiota was dominated by *Pseudomonadaceae*, and tonsil microbiota showed a predominance by *Streptococcaceae*; adenoid microbiota was the most complex, including *Pseudomonadaceae*, *Streptococcaceae*, *Fusobacteriaceae*, and *Pasteurellaceae*, and shared microorganisms found both in tonsils and middle ear, supporting the hypothesis that the adenoid pad could act as a reservoir for both of these sites [68].

Relevant new insights on OME were subsequently provided in an Australian study analyzing NP swabs, MEF, and adenoid specimens from 11 indigenous children undergoing surgery: MEF microbiome was characterized by low diversity indices and predominance of a single bacteria, in most cases *A. otitidis*, *H. influenzae*, or *Streptococcus* spp. In particular, *A. otitidis* was the most common taxa in MEF and was not detected in any NP or adenoid samples. Thus, authors speculated that its origin from NP was unlikely and that it could represent a typical microorganism of the ME niche; however, as *A. otitidis* is a known commensal of the ear canal [69], further studies were warranted to understand its role and the influence of the ear canal flora, especially in children who suffer from recurrent tympanic membrane perforations [70].

Fago-Olsen et al. analyzed microbiota of palatine tonsils and adenoids from children undergoing surgery for adenoid/tonsillar hyperplasia vs. subjects undergoing surgery for secretory otitis media (SOM), showing that several microorganisms were occasionally co-detected in both sites, but *H. influenzae*, *S. pneumoniae*, and *M. catarrhalis* were significantly more abundant in the adenoids and almost absent from palatine tonsils, indicating that adenoids but not palatine tonsils could act as main reservoir of pathogens leading to OM. However, it should be noted that this study did not include MEF microbiota analysis [71].

Data concerning dissimilarities between NP and MEF microbiome were provided in a following investigation including 10 children undergoing adenotonsillectomy and grommet insertion for OME. The authors reported that adenoid and tonsil microbiota shared a higher similarity than adenoid and ME, thus questioning the PRH in OME. According to previous findings, *Alloiococcus* and *Turicella* were detected only in MEF samples; however, the most abundant genera in middle ear were *Fusobacterium* and *Staphylococcus* [72]. These data were subsequently confirmed in an investigation by Ari et al. on a larger population of children with OME: ME microbiome was characterized by a significant predominance of *Alloicoccus otitidis* (44%), *Turicella otitidis* (6%), and *Staphylococcus auricularis* (3%), while adenoid harbored a high relative abundance of *Rothia*, *Staphylococcus*, and *Granulicatella*. As for diversity indices, no significant dissimilarities in alpha-diversity were found between MEF and adenoid niches [73].

The potential role of *A. otitidis* as a key bacteria of the ME was confirmed in an investigation by Chan et al., through the analysis of paired MEF samples and adenoid swabs from children undergoing grommet insertion for OME and of adenoid swabs from healthy subjects. Data evidenced a different composition in microbial communities between paired MEF and adenoid, as 13 of the 17 most abundant genera showed a statistically significant difference in relative abundance. In particular, *A. otitidis* was the predominant OTU in MEF (23% mean relative abundance), while it was almost absent in adenoid samples (<1% relative abundance). Interestingly, this taxa was found in greater abundance in children with unilateral effusion. Authors postulated that the dissimilarities between the MEF and adenoid microbiota could question the PRH in children with OME: Adenoidal hypertrophy and Eustachian tube dysfunction predispose to OME, but subsequent modifications in the ME environment determine an unbalance in the local flora with the predominance of a certain microorganism that can potentially lead to acute disease [74].

Similarly, caution when using nasopharyngeal microbiota as a proxy for ME was warranted by Boers et al. in an investigation comparing NP and ME microbiota in children with gastro-esophageal reflux (GER) associated OM vs. children who suffered from OM without GER. Authors enrolled 30 subjects with RAOM, COME or both undergoing tympanostomy tube placement, identifying *Alloiooccus* spp. and *Turicella* spp. as the most abundant taxa in MEF while absent in NP samples. As for GER, no apparent effects were found on the NP and ME microbiota in the two groups [75].

A more recent investigation conducted in a tertiary hospital in China analyzed ME and adenoid microbiota from children undergoing surgery for OME and adenoid hypertrophy (AH) vs. adenoid microbiota from subjects without ear disease undergoing adenotonsillectomy for OSA. ME was dominated by *Haemophilus* (14.75%), followed by *Staphylococcus* (9.37%) and *Halomonas* (7.85%); moreover, in contrast with previous findings, *Alloiococcus otitidis* had low relative abundance in this site (3.75%), and *Turicella* was not reported at all among the most abundant genera: Authors stated that these differences with previous findings could be attributable to variation in sampling methods, sample size or geographical location. Four taxa were found to be significantly differentially abundant between ME and adenoid in OME group (*Streptococcus*, *Neisseria*, *Alloprevotella*, and *Actinobacillus*), while the classical otopathogens were commonly found both in adenoid and ME in all OME patients. Adenoid microbiota in controls was composed predominantly by *Haemophilus* (15.96%), *Streptococcus* (13.33%), and *Moraxella* (12.28%); however, no significant differences in relative abundances of these genera were found in adenoids of OME patients vs. controls. According to this data and to previous findings, authors concluded that the dissimilarities in microbial compositions between these two niches challenge the PRH in OME [76].

The potential reservoirs for ME microbiome in children with OME were investigated by Chan et al.: MEF analysis showed similar results to the previous studies, as ME was dominated by *A. otitidis*, followed by *Haemophilus*, *Moraxella*, *Staphylococcus*, and *Streptococcus*; the EAC microbiome was mostly constituted by *A. otitidis*, *Staphylococcus* and *Pseudomonas* with rare otopathogens, whereas adenoid microbiome was composed prevalently by otopathogens, with rare EAC genera such as *Alloiococcus*. Basing on this data and on the previous study, authors concluded that both EAC and NP could act as a reservoir for the middle ear in children with OME. However, as bacterial translocation across an intact tympanum has not been demonstrated yet, a membrane perforation (spontaneous or iatrogenic) is probably needed to allow bacteria to translocate from EAC to ME. Unfortunately, a history of previous perforations in this cohort is not available [77].

Another pivotal genera in OME pathogenesis is *Haemophilus*, as highlighted in a study on ME microbiome in 55 children with chronic middle ear effusion: the most abundant genera were *Haemophilus* (relative abundance 22.54%), *Moraxella* (11.11%), *Turicella* (7.84%), *Alcaligenaceae* (5.84%), *Pseudomonas* (5.40%), and *Alloiococcus* (5.08%). Moreover, children were grouped by age, hearing loss, and mucin type expression in MEF: *Haemophilus* was significantly more abundant in children with hearing loss and was associated to MEF containing MUC5B and MUC5A, suggesting a correlation between hearing loss and mucin content in relationship to *Haemophilus* abundance [78].

Kolbe et al. provided data on 50 children undergoing tube placement for COME with a more detailed taxonomic resolution. In contrast to previous data that observed a predominance by *Alloiococcus*, *Moraxella*, or *Haemophilus* in MEF, in this study, microbial communities were highly variable, and the classical otopathogens were absent in about half of the samples. Moreover, authors compared subject based on whether they had a history of lower airway disease (asthma or bronchiolitis): *Haemophilus*, *Staphylococcus*, and *Moraxella* were significantly more abundant in children with lower airway diseases, while *Turicella* and *Alloiococcus* were less prevalent; in addition, ME microbial communities in children with history of asthma/bronchiolitis were significantly less diverse than children who had only COME [79].

Nasopharyngeal microbiome is less diverse in children suffering from OME than in controls, as highlighted by two case-control studies [80,81]. In particular, Walker et al. showed that the nasal microbiome in children with OME is composed of a higher abundance of pathogens, with a lower abundance of commensals as alpha-hemolytic Streptococci and *Lactococcus*. Moreover, cluster analysis revealed that profiles dominated by *Corynebacterium*, *Streptococcus*, or *Moraxella* were associated with COME, while healthy children had a more mixed bacterial profile with higher abundance of commensals [81].

In conclusion, investigations on OME discussed above confirm the role of the known otopathogens, in particular *H. influenzae*, as the predominant taxa in MEF during disease. Moreover, as previously described for AOM, *A. otitidis* and *T. otitidis* are frequently identified as abundant members of the ME microbiota. Studies have so far failed to define the possible reservoir for ME microbiome, and it is not possible to exclude a sample contamination from the EAC, especially in a low biomass environment as the ME. Concerning this theme, we believe that further studies should also be focused on patients with a history of tympanic membrane perforation, which might be the entryway for microorganisms that colonize the EAC.

An overview on microbiome study in OME previously discussed is reported in Table 3.

## 6. Chronic Suppurative Otitis Media

Chronic suppurative otitis media (CSOM) is defined as a chronic inflammation of the middle ear and mastoid cavity, with recurrent or persistent ear discharge through a non-intact tympanic membrane [10]. Less evidence is available on microbial communities in pediatric patients suffering from this condition.

Neef et al. compared 24 children with CSOM undergoing mastoid surgery to 22 healthy controls undergoing ear surgery for other conditions as cochlear implantation or benign brain tumor removal. Microbiota analysis and conventional culture were performed on swabs collected from middle ear and mastoid cavity during surgery. Authors did not observe a typical bacterial profile associated to CSOM, but highlighted the limits of the conventional culture-based approach, as no bacteria were detected by culture in healthy subjects. By contrast, molecular analysis detected potential pathogens as *Staphylococcus*, *Pseudomonas*, and *Haemophilus* even in healthy controls. As for diversity, authors observed a major inter-personal difference among CSOM patients, whereas this finding was not observed for controls. This data supported the hypothesis that microbial communities’ disruption and dysbiosis could be implicated in CSOM pathogenesis [82].

These dissimilarities among patients suffering from CSOM are age-related, as reported by Minami et al. In this investigation, middle ear swabs were collected during surgery in pediatric and adult patients undergoing tympanoplasty for wet or dry COM vs. subjects undergoing surgery from other conditions than otitis media. *Proteobacteria* was the predominant phylum detected in normal subjects, both adults and children. However, the normal middle ear microbiota differed significantly according to age: Authors concluded that this dissimilarity between adults and children could be related to the higher incidence of *Staphylococcus* (*Firmicutes* phylum) in adults. Subjects with active inflammation and wet COM had a lower abundance of *Proteobacteria* and a higher incidence of *Firmicutes*: Authors warranted this finding to be considered in the pathogenesis of active inflammation in COM, in relation to the potential penetration of several exogenous pathogens through a chronic perforation. On the other hand, microbiome of dry COM was not significantly different from normal middle ear [83].

Santos-Cortez et al. previously performed an investigation comparing ME and EAC microbiome in 16 indigenous Filipino subject with chronic tympanic membrane perforation, showing that the microbial communities between these two niches were similar, probably due to a cross-contamination process through the perforated eardrum. Moreover, authors investigated microbiota composition in subjects who were carrier of the A2ML1 gene, which encodes an alpha-2 macroglobulin-like 1 protein, previously identified as a genetically determined risk factor for of otitis media [84]. Authors detected a higher relative abundance of *Fusobacterium*, *Porphyromonas*, *Peptostreptococcus*, *Parvimonas*, and *Bacteroides* in the ME of A2ML1-carrier patients, while *Alloiococcus*, *Staphylococcus*, *Proteus*, and *Haemophilus* were more abundant in ME of non-carrier subjects. Authors speculated that the expected loss-of-function of A2ML1 protein could influence ME microbiota composition promoting survival and growth of specific microorganism. This findings warrant further investigations on the relationship between host genotype and microbiota in OM [85].

Evidence on CSOM is lacking and does not show peculiar features of microbial communities in this OM phenotype. Moreover, investigations discussed above include both adults and children, thus it is difficult to draw any general conclusion in the pediatric population. The penetration of microorganisms residing in the EAC from the chronic tympanic membrane perforation has been considered in the pathogenesis of the active inflammation in CSOM, but further studies are needed to define with major detail this aspect.

An overview on microbiome study in CSOM previously discussed is reported in Table 4.

## 7. Probiotic Therapy

Prevention of OM in children represents one of the most difficult aspects in the clinical management of these patients.

Restoration of dysbiosis through administration of probiotic strains is a preventive strategy that has gained major clinical and scientific interest in recent years in several diseases, including otitis media.

Probiotics are defined as “live microorganisms that, when administered in adequate amounts, confer a health benefit on the host” [86]. The introduction of high-throughput sequencing methods has allowed the investigation of entire bacterial communities and the identification of microorganisms associated to health status in various conditions.

As previously discussed, evidence on microbiota in children suffering from OM suggest that *Corynebacterium* spp. and *Dolosigranulum pigrum* are potential keystone taxa in the URT; thus, major interest has been directed towards these two microorganisms and their potential use as probiotics.

A detailed discussion of evidence available on probiotic therapy in OM goes beyond the scope of this review, as it has been recently extensively reviewed elsewhere.

A recent review by van den Broek et al. described novel insights on probiotic therapy in OM [87]. Basing on Koch’s postulates, authors introduced the “probiotic postulates” to define the ideal probiotic strain to be used in clinical practice: The microorganism can be found in high abundance in health status and decreased abundance during disease; the microorganism can be isolated from a healthy organism and grown in pure culture; the cultured organism should promote health when introduced into a diseased organism; it should be possible to re-isolate these microorganisms as identical to the original agent from the healthy host. According to available evidence and to this postulates, authors identified *Dolosigranulum* as a prime candidate for the development of probiotic therapy.

However, current knowledge is still not sufficient to define probiotic efficacy for preventing OM. A recent systematic review included 13 studies on this subject, concluding that available evidence on probiotics use for the prevention of AOM is limited; among the various formulations, possible benefit could derive from nasal administration [88].

The most important limitations in evidence on this topic are poor to moderate quality of the investigations and great heterogeneity in route of administration (oral vs. intranasal), probiotic strains included in formulations, duration of therapy, and outcome measures.

## 8. Conclusions

The introduction of the modern molecular techniques and the subsequent investigations on microbial communities in the human organisms have changed our conceptions of health and disease and our approach to infectious conditions.

It is indeed well known that health and disease status are not merely determined by the presence or the absence of a pathogen but depend on a complex balance established among pathogens, resident microbiota, and host immune response.

Investigations previously described in this review have provided novel insights on the pathogenesis of middle ear diseases and led to the identification of both possible new causative agents and of potential protective bacteria, showing that imbalances in bacterial communities of the URT and ME could influence the natural history of otitis media in children.

However, scientific data on this topic are often difficult to compare because of methodological differences in specimen collection and analysis, in the site of investigation, and in data reporting. Moreover, a lack of standard diagnostic criteria for OM across countries often influences the enrollment phase and contributes to increase the heterogeneity among populations under investigation.

Another element that complicates data interpretation and deserves standardization is the use of different databases during OTUs assignment. This is a relevant issue that should be taken into consideration for two main reasons: different databases might lead to heterogeneous results; some taxa could be misclassified with certain databases, as reported for *A. otitidis* and *T. otitidis* [89].

We believe that future investigation should be focused on the following aspects:

- Defining standard criteria of specimen collection, analysis, and data reporting, in order to facilitate data comparison across studies;
- Deepening our knowledge on the impact of various exogenous factors that have been less explored, such as active/passive smoking, vaccines, and viral infections;
- Confirming the role of *Corynebacterium* and/or *Dolosigranulum* as keystone taxa, in order to evaluate their possible use as probiotics;
- Understanding the development of URT and ME microbiota at different ages, in order to identify a potential “window of opportunity” in which therapeutic interventions as probiotic administration could be more effective, before the establishment of a stable microbial community that could be modulated with difficulty;
- Investigating the concordance between NP and ME microbiota, in order to better define the role of adenoid pad as a proxy for ME;
- Providing data on microbial communities in ME, which is no longer considered a sterile site;
- Defining with major detail the features of NP and ME microbial communities in different OM phenotypes, in particular in children with recurrent STMP.

## Figures and Tables

**Table 1:** Definitions of common terms used in microbiota investigations.

**Microbiota**	Ecological communities of commensal, symbiotic and pathogenic microorganisms that colonize several body sites, as the gastrointestinal tract, respiratory system, oral cavity, skin, and female reproductive system
**Microbiome**	Genetic material of the microorganisms of a community
**Keystone Species**	Microorganisms with a great impact on an ecological community, considered important in maintaining its organization and function
**Biodiversity**	Number of OTUs in a community and their relative abundance. It is determined by richness (how many OTUs in a sample?) and evenness (how equally distributed relative abundances are in a sample?)
**Alpha-Diversity**	Diversity within sample: how abundant OTUs are in relation to others in the same sample?
**Beta-Diversity**	Measure that compares different microbial communities
**Operational Taxonomic Unit (OTU)**	Cluster of related sequences (usually with 97% or more similarity) that represent a taxonomic unit of a microorganism

**Table 2:** Overview of investigations on microbiota and acute otitis media discussed in this review.

Title (Year of Publication) [Ref]	Study Design	N. of Subjects	Age	Site of Investigation	Main Findings
Microbial Communities of the Upper Respiratory Tract and Otitis Media in Children (2011) [20]	Comparison of NP microbial communities in children with and without OM	108 (25 with AOM; 83 without AOM)	6–78 m	NP	Microbial communities with *S. pneumoniae* were significantly less diverse and less even Higher relative abundance of *Corynebacterium* and *Dolosigranulum*, in addition to *Propionibacterium*, *Lactococcus*, and *Staphylococcus*, was associated with a lower incidence of pneumococcal colonization and lower risk of AOM
Nasopharyngeal Microbiota in Infants with Acute Otitis Media (2012) [44]	Comparison of NP microbial communities in children with and without OM	163 (153 with AOM; 10 without AOM)	<2 y	NP	NP bacterial density was lower during an AOM episode in comparison to health Otopathogens predominated over commensal families during AOM
Upper Respiratory Tract Microbial Communities, Acute Otitis Media Pathogens, and Antibiotic Use in Healthy and Sick Children (2012) [19]	Comparison of NP microbial communities in healthy children vs. children with URTI with and without concurrent AOM	240 (73 healthy subjects; 95 subjects with URTI without concurrent AOM; 72 subjects with URTI with concurrent AOM)	6 m–3 y	NP	Lower diversity was associated with a higher colonization rate by *S. pneumoniae*, *H. influenzae*, and *M. catarrhalis* Biodiversity levels were significantly higher in healthy children than during disease Children with antibiotic use in the past 6 months and a higher abundance of *Lactococcus* and *Propionibacterium* had a lower risk of AOM Children with no antibiotic use in the past 6 months, a low abundance of *Streptococcus* and *Haemophilus*, and a high abundance of *Corynebacterium* and *Dolosigranulum* had a lower risk of AOM
Nasopharyngeal microbiota in infants and changes during viral upper respiratory tract infection and acute otitis media (2017) [45]	NP microbiota analysis of children followed from near birth for the first 12 months of life or until the occurrence of the first AOM episode. NP swabs collected monthly or during each URTI or AOM episode.	139 patients (971 samples)	<1 y	NP	Bacterial diversity was lower in culture-samples positive for *S. pneumoniae* and *H. influenzae* compared to cultured-negative samples Otopathogen colonization was related to higher incidence of URTI Higher abundance of otopathogens and lower abundance of *Pseudomonas*, *Myroides*, *Yersinia*, and *Sphingomonas* during URTI and AOM Higher otopathogen abundance during symptomatic viral infection but not during asymptomatic infection An unstable microbiota during URTI and the predominance of otopathogens was associated with a higher risk of transition from URTI to AOM
The Adenoid Microbiome in Recurrent Acute Otitis Media and Obstructive Sleep Apnea (2017) [58]	Comparison of adenoid microbiota in subjects undergoing surgery for RAOM or OSA	10 (5 AOM; 5 OSA)	2–11 y	Adenoid	*H. influenzae*, *M. catarrhalis*, *S. pneumoniae*, *P. aeruginosa*, and *S. aureus* were predominant in all samples Relative abundance of *S. pneumoniae* and *M. catharralis* was higher in the RAOM group The microbial profiles associated with RAOM were different from, but overlapped with OSA
Next-Generation Sequencing Combined with Specific PCR Assays To Determine the Bacterial 16S rRNA Gene Profiles of Middle Ear Fluid Collected from Children with Acute Otitis Media (2017) [59]	ME microbiota analysis during AOM episodes	79 subjects (90 samples)	5–42 m	ME	*S. pneumoniae* was detected in 31% of samples, *H. influenzae* in 27%, *M. catarrhalis* in 20%, *Staphylococcus* spp. in 23%, *T. otitidis* in 5.6%, *A. otitidis* in 3.3% *S. pneumoniae* was the dominant pathogen in 16% of samples, *H. influenzae* in 17%, *M. catarrhalis* in 5.6%
A microbiome case-control study of recurrent acute otitis media identified potentially protective bacterial genera (2018) [22]	Comparison of NP microbiota between children undergoing grommet insertion for RAOM (cases) vs. healthy children (controls); analysis of ME and EAC microbiota in cases	196 (93 cases; 103 controls)	<5 y	NP ME EAC	Significantly higher abundance of *Corynebacterium* and *Dolosigranulum* was detected in NP of controls in comparison to cases Paired NP and ME were not highly concordant: *Alloiococcus*, and *Turicella* were abundant in ME and EAC of cases and almost absent in NP of both groups *Gemella* and *Neisseria* were typical of the NP in cases prevalent in the middle ear
Comparative Analysis of Microbiome in Nasopharynx and Middle Ear in Young Children with Acute Otitis Media (2019) [60]	Comparison of NP microbiota 1 to 3 weeks prior to onset of AOM vs. at onset of AOM; comparison of NP and ME microbiome during AOM	6	6–24 m	NP ME	Significantly higher abundance of *A. otitidis* detected in MEF during AOM compared to NP in health and disease NP microbiome during health had a significantly higher diversity than during AOM
Age-Dependent Dissimilarity of the Nasopharyngeal and Middle Ear Microbiota in Children with Acute Otitis Media (2019) [61]	NP microbiota analysis during AOM; Paired NP and ME microbiota analysis in children with STMP	286 (42/286 MEF from STMP)	0–6 y	NP ME	Alpha and beta diversity levels were strictly related to age: older children had a higher richness and more personalized bacterial profiles NP and MEF microbiome were concordant when MEF was dominated by *S. pyogenes*, *H. influenzae*, or *S. pneumoniae*
Respiratory Microbiota Predicts Clinical Disease Course of Acute Otorrhea in Children with Tympanostomy Tubes (2019) [67]	Paired analysis of NP and ME microbiota in children with otorrhea on tympanostomy tubes	94	<5 y	NP ME	Microbiota composition of NP and ME differed significantly, although paired NP and ME samples were more similar than unpaired samples *P. aeruginosa*, *S. aureus*, *S. pyogenes*, *T. otitidis*, *K. pneumoniae*, and *Haemophilus* spp. were correlated between NP and ME *Moraxella* spp., *S. pneumoniae*, and *Corynebacterium/Dolosigranulum* were predominant in NP than in MEF *Turicella*, *P. aeruginosa*, and *S. aureus* were strongly associated to ME Higher abundance of *Corynebacterium* and *Dolosigranulum* in NP related to better clinical outcomes

AOM: Acute otitis media. OSA: Obstructive Sleep Apnea. RAOM: Recurrent Acute Otitis media. EAC: External Auditory Canal. NP: Nasopharynx. STMP: Spontaneous Tympanic Membrane Perforation. ME: Middle Ear. MEF: Middle Ear Fluid. URTI: Upper Respiratory Tract Infection.

**Table 3:** Overview of investigations on microbiota and otitis media with effusion discussed in this review.

Title (Year of Publication) [Ref]	Study Design	N. of Subjects	Age	Site of Investigation	Main Findings
The Otologic Microbiome: A Study of the Bacterial Microbiota in a Pediatric Patient with Chronic Serous Otitis Media Using 16SrRNA Gene-Based Pyrosequencing (2011) [68]	Microbiota analysis in ME, adenoid, and tonsil specimens from one pediatric patient with chronic serous otitis media undergoing adenotonsillectomy and bilateral tympanic tube insertion	1	8 y	Adenoid ME Tonsil	*Pseudomonadaceae* were predominant in ME *Streptococcaceae* were predominant in tonsil Adenoid microbiota included multiple predominant bacteria: *Pseudomonadaceae*, *Streptococcaceae*, *Fusobacteriaceae*, and *Pasteurellaceae* Adenoid microbiota shared bacteria found both in tonsils and middle ear
The microbiome of otitis media with effusion in Indigenous Australian children (2015) [70]	MEF, NP, and adenoid microbiota analysis in children undergoing surgical treatment for OME	11	3–9 y	NP Adenoid ME	ME microbiota was dominated by *A. otitidis*, *H. influenzae*, or *Streptococcus* spp. *A. otitidis* was the most common OTU in MEF and was not detected in any NP or adenoid samples *Streptococcus* spp., *H. influenzae*, and *M. catarrhalis* were common to all sample types
The Microbiome of Otitis Media with Effusion (2016) [74]	ME and adenoid microbiota analysis in children undergoing adenoidectomy with ventilation tube insertion for chronic OME. Adenoid microbiota analysis from healthy subjects enrolled as controls	33 (23 subjects with OME; 10 healthy controls)	OME group: 1–8 y Control group: 1–12 y	ME Adenoid	ME microbiota was dominated by *A. otitidis* (23% mean relative abundance), *Haemophilus* (22%), *Moraxella* (5%), and *Streptococcus* (5%) Different microbial composition between paired MEF and adenoid: 13/17 of the most abundant genera showed a statistically significant difference in relative abundance *A. otitidis* was the predominant OTU in MEF (23% mean relative abundance), while it was almost absent in adenoid samples (<1% relative abundance)
The Relationship of the Middle Ear Effusion Microbiome to Secretory Mucin Production in Pediatric Patients with Chronic Otitis Media (2016) [78]	Microbiota analysis and mucin detection in MEF collected from children undergoing myringotomy with tympanostomy tube placement for chronic OME	55	3–176 m	ME	The most abundant genera were *Haemophilus* (relative abundance 22.54%), *Moraxella* (11.11%), *Turicella* (7.84%), *Alcaligenaceae* (5.84%), *Pseudomonas* (5.40%), and *Alloiococcus* (5.08%) *Haemophilus* was significantly more abundant in children with hearing loss and was associated to samples containing secretory mucins as MUC5B and MUC5A
Identification of the Bacterial Reservoirs for the Middle Ear Using Phylogenic Analysis (2017) [77]	ME and EAC microbiota analysis in children undergoing surgery for OME. Adenoid pad and ME microbiota analysis data were included from a previous study	18	1–14 y	ME EAC	The MEF microbiota was dominated by *A. Otitidis* (37.5%), *Haemophilus* (14.4%), *Moraxella* (10.0%), *Staphylococcus* (8.2%), and *Streptococcus* (3.8%) The EAC had a high abundance of *Alloiococcus* (58.0%), *Staphylococcus* (20.8%), and *Pseudomonas* (3.2) with rare otopathogens The adenoid microbiota had a high abundance of otopathogens with rare EAC genera: *Alloiococcus* (0.1% vs. 28.9%), *Haemophilus* (25.2% vs. 18.2%), *Staphylococcus* (0.2% vs. 10.8%), *Streptococcus* (12.7% vs. 4.2%), and *Pseudomonas* (0 vs.2.1%, respectively)
Pathogen reservoir hypothesis investigated by analyses of the adenotonsillar and middle ear microbiota (2018) [72]	Adenoid, middle ear, and tonsil microbiota analysis in children undergoing surgical treatment for OME	10	5–10 y	Adenoid ME Tonsil	The most abundant genera in all sites were *Fusobacterium*, *Haemophilus*, *Neisseria*, and *Porphyromonas* Higher proportion of *Haemophilus* and *Moraxella* in the adenoid than ME *Alloiococcus* and *Turicella* were detected only in MEF samples Adenoid and tonsil microbiota shared a higher similarity than adenoid and ME
Characterization of the nasopharyngeal and middle ear microbiota in gastroesophageal reflux-prone versus gastroesophageal reflux non-prone children (2018) [75]	Analysis of NP and ME microbiota in children suffering from GER-associated OM vs. OM only undergoing surgical treatment for RAOM, COME, or both	30 (9 subjects with GER-associated OM; 21 subjects with OM without GER)	GER group 1.3–6 y No GER group 0.8–12–8 y	NP ME	No effect of GER on NP and ME microbiota in the two groups *Alloiococcus* spp. and *Turicella* spp. were the most common taxa in MEF and were not detected in any NP swab
The Adenoids but Not the Palatine Tonsils Serve as a Reservoir for Bacteria Associated with Secretory Otitis Media in Small Children (2019) [76]	Adenoid and tonsillar microbiota analysis in children undergoing surgical treatment for hyperplasia of adenoids/tonsils without infection (HP group) vs. children undergoing surgery for SOM	28 (112 samples) (14 subjects in HP group; 14 subjects in SOM group)	HP group 24–65 m SOM group 15–59 m	Adenoid Tonsils	The number of OTUs detected in the adenoids from the HP group was significantly lower compared to the number detected in adenoids from SOM group Streptococcus was the most abundant genus (average 25.6%) followed by *Fusobacterium* (11.1%) and *Haemophilus* (10.3%) Microbial communities were significantly different between the adenoid and tonsil samples *S. pneumoniae* was significantly more abundant in the adenoids of HP group compared to adenoids of SOM group *Fusobacterium nucleatum* was abundant in the adenoids of HP group but was almost in the adenoids of SOM group The classical otopathogens (*H. influenzae*, *S. pneumoniae*, and *M. catarrhalis*) were significantly more abundant in the adenoids than in the tonsils
Nasal microbial composition and chronic otitis media with effusion: A case-control study (2019) [81]	Comparison of nasal microbiota between children undergoing surgery for COME vs. healthy subjects	178 (73 cases; 105 controls)	Case group: mean age 47.5 m Control group: mean age 49.6 m	Nasal (anterior nares)	Children with COME had lower diversity than healthy controls Children with COME had a higher abundance of otopathogens and lower abundance of commensals as Haemolytic Streptococci and *Lactococcus* Profiles that were *Corynebacterium-dominated* or *Moraxella-dominated* were associated with COME
Altered Middle Ear Microbiome in Children with Chronic Otitis Media with Effusion and Respiratory Illnesses (2019) [79]	Comparison of ME microbiota children with chronic OME and history of lower airways disease (asthma or bronchiolitis) vs. children with chronic OME without history of lower airways disease	50 (13 with history of lower airway disease)	3–176 m	ME	The ME microbiome was significantly less diverse in children with lower airway disease *Haemophilus*, *Staphylococcus*, and *Moraxella* were significantly more abundant in ME of children with lower airways disease
Analysis of the Microbiome in the Adenoids of Korean Children with Otitis Media with Effusion (2019) [80]	Adenoid microbiota comparison between children undergoing surgery for OME vs. children without undergoing surgery for obstructive symptoms	32 (16 subjects with OME; 16 subjects without OME)	19 m–15 y	Adenoid	Diversity levels were lower in the OME group *Haemophilus* was the most abundant in the OME group *Prevotella*, *Delftia*, and *Corynebacterium* were the dominant genera in the OME group
The bacteriome of otitis media with effusion: does it originate from the adenoid? (2019) [73]	Adenoid and ME microbiota analysis in children undergoing surgery for OME	25	1.5–9 y	Adenoid ME	ME microbiome was dominated by *A. otitis* (44%), *T. otitidis* (6%), and *S. auricularis* (3%) Adenoid microbiome was dominated by *Rothia*, *Staphylococcus*, and *Granulicatella* No statistically significant difference in alpha diversity between the two niches; adenoid samples clustered in the beta diversity graph
The microbiomes of adenoid and middle ear in children with otitis media with effusion and hypertrophy from a tertiary hospital in China (2020) [76]	Adenoid and ME microbiota analysis in children undergoing surgical treatment for OME vs. adenoid microbiota analysis in children undergoing surgery for OSA without ear disease	30 (15 in OME group; 15 in OSA group)	OME group 60–108 m OSA group 8–96 m	Adenoid ME	ME in OME was dominated by *Haemophilus* (14.75%), *Staphylococcus* (9.37%), and *Halomonas* (7.85%) Low abundance of *A. otitis* (3.75%) in ME in OME group Adenoid microbiota in OME group was dominated by *Haemophilus* (21.87%), *Streptococcus* (19.65%), and *Neisseria* (5.8%) Adenoid microbiota in OSA was dominated by *Haemophilus* (15.96%), *Streptococcus* (13.33%), and *Moraxella* (12.28%) No significant differences in alpha-diversity between ME and adenoids in OME group Beta diversity analyses showed that the microbiome structure of ME was dissimilar the adenoid one in OME patients: taxa found to be significantly differentially abundant between these two sites were *Streptococcus, Neisseria*, *Alloprevotella*, and *Actinobacillus*

OME: Otitis Media with Effusion. COME: Chronic Otitis Media with Effusion. NP: Nasopharynx. ME: Middle Ear. MEF: Middle Ear Fluid. GER: Gastro-esophageal reflux. RAOM: Recurrent acute otitis media. SOM: Secretive otitis media. EAC: External auditory Canal. OTU: Operational Taxonomic Unit. OSA: Obstructive sleep apnea.

**Table 4:** Overview of investigations on microbiota and chronic suppurative otitis media discussed in this review.

Title (Year of Publication) [Ref]	Study Design	N. of Subjects	Age	Site of Investigation	Main Findings
Molecular Microbiological Profile of Chronic Suppurative Otitis Media (2016) [82]	Comparison of ME and mastoid microbiota in patients with CSOM undergoing surgery vs. healthy controls	46 (24 subjects with CSOM; 22 healthy subjects)	6 m–85 y	ME Mastoid cavity	No typical bacterial profile associated to CSOM No bacteria were detected by culture in healthy subjects, while molecular analysis detected potential pathogens such as *Staphylococcus*, *Pseudomonas*, and *Haemophilus* Inter-personal difference in diversity levels among CSOM patients but not among controls
Microbiomes of the Normal Middle Ear and Ears with Chronic Otitis Media (2017) [83]	ME microbiota analysis in patients undergoing tympanoplasty for wet or dry COM vs. subjects undergoing surgery from other conditions than otitis media	155 (67 healthy subjects; 44 subjects with COM without active infection; 44 subjects with COM with active infection)	1–84 y	ME	The normal middle ear microbiota differed significantly according to age: in particular, a higher incidence of *Staphylococcus* (*Firmicutes* phylum) was detected in adults Microbiome of dry COM was not significantly different from normal middle ear Lower abundance of *Proteobacteria* and higher incidence of *Firmicutes* in subjects with active inflammation and wet COM
Middle ear microbiome differences in indigenous Filipinos with chronic otitis media due to a duplication in the A2ML1 gene (2016) [85]	ME and EAC microbiota analysis in indigenous Filipinos with chronic otitis media; comparison of microbial communities in subjects carriers of A2ML1 variant vs. non carrier subjects	16 (11 subjects carriers of A2ML1 variant)	4–24 y	ME EAC	Microbial communities between ME and EAC were similar Higher relative abundance of *Fusobacterium*, *Porphyromonas*, *Peptostreptococcus*, *Parvimonas*, and *Bacteroides* in the ME of A2ML1-carrier patients Higher relative abundance of *Alloiococcus*, *Staphylococcus*, *Proteus*, and *Haemophilus* in ME of non-carrier subjects

CSOM: Chronic Suppurative Otitis Media. COM: Chronic Otitis Media. ME: Middle Ear. EAC: External auditory canal.
